# Cleaning a dark matter detector: A case of ontological and normative elusiveness

**DOI:** 10.1177/03063127251361158

**Published:** 2025-08-30

**Authors:** Jaco de Swart, Annemarie Mol

**Affiliations:** 1 Program in Science, Technology, and Society, and Department of Physics, Massachusetts Institute of Technology, Cambridge, MA, USA; 2 Department of Anthropology, Amsterdam Institute for Social Science Research, University of Amsterdam, Amsterdam, The Netherlands

**Keywords:** clean, dark matter, ontologies, normativities, environment, laboratory, experiments, particle physics

## Abstract

Laboratory sciences crucially depend on the cleanliness of experiments. But what is clean? In this article, we show that the salience of the valuation *clean* emerges through its relation to a particular ontological repertoire. Our case is the XENONnT experiment in the Gran Sasso Mountains of Italy, designed to detect dark matter in the form of hypothetical WIMPs (Weakly Interacting Massive Particles). In this experiment, dirt presents a significant disruption, as contaminations can mimic the signals of WIMPs, and electronegative molecules risk erasing such signals. The ideosyncratic cleanliness required makes the practice of cleaning the XENONnT detector exceedingly difficult. So far, the ontological question ‘do WIMPs exist?’ remains open, which means that the normative question ‘is the detector clean enough?’ cannot be answered either. In addition, more cleaning will make the detector sensitive to a background of unremovable neutrinos—hence irredeemably dirty. With the normative goal of a ‘clean detector’ out of reach, the ontological question ‘do WIMPs exist?’ is bound to remain open as well. Alternative experiments therefore hunt for different hypothetical dark matter candidates, with different equipment, requiring different kinds of cleanliness. At the same time, the XENONnT experiment must navigate tensions between its own cleanliness goals and rules meant to ensure the environmental cleanliness of the Gran Sasso National Park. Cleaning turns out to be dirty. This leads us to ask: Which *goods* deserve to be cherished, and, intertwined with that, which *realities* deserve to be cared for?

‘You need to imagine we clean everything’, Tobias told us. ‘Each single screw in this detector went through the hands of the cleaning team, everything.’ Tobias—or that is how we call him here—worked for the XENONnT experiment that sought to detect dark matter in the form of Weakly Interacting Massive Particles, WIMPs. While the existence of dark matter has been a central hypothesis in cosmology for over four decades, the nature of this hypothetical dark matter is still elusive. WIMPs could be the answer. The XENONnT collaboration was fueled by the hope that they would be able to detect these WIMPs. It was Tobias’s role in this endeavor to coordinate the so-called ‘cleaning campaign’. Pablo, who was involved in this campaign as a PhD student, related, half laughing: ‘I heard that people were working on cleaning and I was like “what does that mean?” The detector? Is that, like, wiping around it? I had no idea. But it’s, literally, all the small parts that will go into the detector. They are all being cleaned, individually. Tedious work.’

In this article, we examine that work. Drawing on long-term participation in physics departments, analytic readings of the relevant literature, and targeted interviews with a wide range of directly involved actors, we outline what cleaning the XENONnT detector entails: how it is done, where it leads, where it halts, which alternatives arise, and what its side effects are. This exploration is motivated by an interest in the intricate *good* of *cleanliness.* Taking inspiration from other inquiries into practices of valuing (e.g., Dussauge et al., 2015; Heuts & Mol, 2013; Vatin, 2013), we hoped that unpacking what cleanliness entails in the case of dark matter research could yield interesting insights. And so it does—as you will see below.

Our efforts also build on the tradition of laboratory studies. Earlier authors in this field have shown that modern sciences are seriously invested in cleanliness. They have pointed out that keeping one’s laboratory clean tends to be one of the preconditions for acquiring knowledge. In this way, they have expanded epistemology from the realm of theoretical prerequisites to that of material arrangements. We take a step further in our inquiry into cleanliness, by putting ontologies—in the plural—at stake (e.g., Law, 2015; Mol, 2002; Thompson, 2007; Woolgar & Lezaun, 2013). Here’s the crux: what exactly ‘clean’ entails varies across ontological repertoires. In other words, it depends on which entities are locally enacted as—potentially—real, and as theoretically and/or practically relevant, as well as on the techniques used to research or otherwise engage with them (Mol, 2012). It is possible to illustrate what this entails by drawing on the earlier laboratory studies, even if they don’t pursue this point. For instance, Gieryn (2018) shows that when geochemists want to study if lead levels in rocks have increased over time, their cleanroom needs to be free of lead. Knorr Cetina (1999) explains that particle collider experiments searching for theoretically predicted phenomena risk ‘that their signs marking interesting events are muffled and smeared by signs from other occurrences in the detector’ (p. 49).^1^

The locally salient *real—*that is, the ontological repertoire at play—determines what is of interest, what is irrelevant, and what counts as a disturbance. Disturbances are to be avoided or eliminated: This is the work of cleaning. Thus, it turns out that clean is both remarkably stable and strikingly diverse: stable, as time and time again *clean* indexes some sort of purity; but also diverse, as from one setting to the next, another type of *dirt*, is to be avoided or eradicated. With what aim exactly? For many scholars in social sciences and humanities, the term clean calls to mind a version of tidiness—an expression of categorical and/or material order, as per Douglas’s (1966) famous dictum, ‘Dirt is matter out of place.’ When we presented our case to colleagues, invariably someone would come up with this quote, as if it were a mantra. And sometimes it fits, as in Mody’s (2001) analysis of material science’s investments in producing ordered structures of atoms, with pollution potentially breaking the order.^2^ However, as we show in what follows, cleanliness is not necessarily about *order*, it may also be about *function*. It may be the kind of purity that allows something—be it a mechanism, an experiment, or an organism—to work, as one of our informants put it (see below). To condense this, we dreamt up an alternative catchphrase, more fitting to situations in which function is at stake: ‘Dirt is a disruptor.’^3^

And this leads on to the first reason that the XENONnT dark matter detector forms such an interesting case: It is easily disrupted and its cleanliness is exceedingly difficult to achieve. While earlier laboratory studies lauded cleanliness as a prerequisite to the production of knowledge, they left the efforts put into cleaning (the dirty work!) unexplored. When in our case we opened them up, these efforts turned out to be impressive. This is because even minute amounts of radioactive particles must be excluded for a xenon detector to function properly, as they would produce signals that mimic those of the WIMPs. Added to that, electronegative molecules as common as oxygen act as dirt because they can absorb WIMP-signals. The version of *clean* pertinent to the detector is very different from alternative versions relevant elsewhere. This discrepancy made it difficult to procure the required building materials and turned determining the best cleaning procedures into an uphill battle.

Did the ‘cleaning campaign’ that Tobias coordinated succeed? Nobody knows. This is one of these moments when facts do not translate into values, when quantities fall short of capturing qualities. It was possible to quantify parameters of cleanliness relevant to the detector—and in terms of the amounts of radioactivity left, the detector was, as our informants put it, ‘the cleanest place on earth’. Yet did this make the detector *adequately* clean, clean enough to fulfill its purpose of detecting dark matter?

This question cannot be answered, because the hypothetical WIMPs have not been found. For the experimenters, this is a tragedy. For us it forms a further interesting aspect of our case: Here we have a situation where WIMPs are potentially real, but cannot be found.^4^ And with the ontological question (do WIMPs exist?) remaining open, the normative question (was the detector clean enough?) cannot be answered either. Hence, the existence of WIMPs and the cleanliness of the detector remain elusive together. The added, yet more tragic and intriguing, layer is that additional cleaning does not help. Instead, it makes the detector sensitive to neutrinos—tiny particles released in nuclear processes (for instance those in the sun). At this point, neutrino signals start to hide the signals of any possible WIMPs. And however coveted detecting neutrinos may be elsewhere, in a detector meant to detect dark matter, they figure as dirt. Hence, as neutrinos come into the picture, the xenon detector shifts from exceedingly clean into irredeemably dirty. A stalemate ensues: with the normative goal (clean enough) remaining out of reach, the ontological question (do WIMPs exist?) cannot be answered.

The ensuing limbo has made some dark matter researchers doubt whether xenon detection of WIMPs is actually the way to go. They have begun to search for dark matter candidates other than WIMPs—notably *axions* and *primordial black holes*. These are embedded in other ontological repertoires and the equipment used to detect them depends on other kinds of cleanliness. Neither the radioactivity nor the electronegative molecules that plague a xenon detector disturb these alternative experiments. This raises wider questions about the relations between different versions of cleanliness (what counts as clean) and between different practices of cleaning (what is done to clean). Although our singular case does not resolve these questions, it does offer an interesting glimpse into the relationships between different versions of *clean*. The ‘cleaning campaign’ did not just depend on the efforts of Tobias and his team but also involved a lot of materials. Water, acids, wipes, protective suits, gloves, and so on. Once such materials were no longer useful, they were discarded in accordance with local environmental regulations—created after previous spills of toxic fluids. This means that the discards of the XENONnT experiment did not pollute the nature reserve in which the laboratory is located. However, in some other environments these discards will inevitably figure as dirt, hindering the functioning of living organisms.

Materials used to achieve internally salient forms of cleanliness manifest themselves externally as environmental pollution. This is not unique to laboratories; it also happens in factories, businesses, offices, hospitals, households, and so on. This leads us to end with a question: How can we better deal with the tense relations between different versions of cleanliness? Which reality do we care to care for?

## It’s not a job. It’s a quest

The XENON experiment that forms our case is one of several experiments searching for dark matter. Current theories hold that 85% of the mass in the universe comes in the form of dark matter—*dark* because so far it has remained invisible, *matter* because it should have a gravitational pull. As physicist Aprile (2021a), director of the XENON experiment, explained in a lecture, ‘Evidence has mounted that the gravitational pull necessary to keep galaxies to stay intact … requires much more matter than what we can see—matter that has eluded the most advanced telescopes to date’. Aprile declared: ‘It is the dominant form of matter in the universe, and yet its nature remains a mystery.’ This mystery is one of the most burning, Nobel prizeworthy, outstanding questions in physics and astronomy. It would be solved by finding dark matter particles and so far (or until recently—see below) WIMPs have been the favorite candidate.^5^ WIMPs are allegedly *massive*, they interact with other particles via gravity, and *weakly interactive*, their probability of bumping into other particles is low. Exactly how massive WIMPs might be, and how much, or little, chance they have of bumping into other particles (physicists call this their *cross-section*), are the questions driving the research.

**Figure 1.**
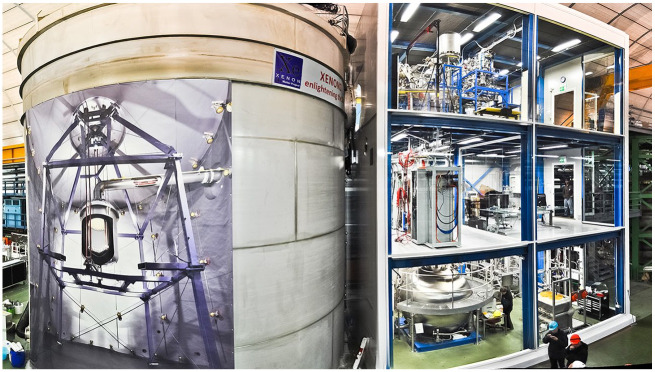
The XENON experiment facility at the underground Gran Sass National Laboratory. The poster on the tank on the left shows the inside of the tank. *Source:* Photo by Roberto Corrieri and Patrick De Perio.

The idea that WIMPs might be the answer to the dark matter question arose in the 1980s (de Swart, 2024; de Swart et al., 2017). Particle physicists were quick to propose that, if WIMPs exist, they could be detected on Earth using a sufficiently sensitive detector. Hence, physicists started thinking about creating such detectors. At first, they used existing particle detectors, such as crystals cooled to very low temperatures. The hope was that WIMPs would collide with the nuclei of the crystals and it would be possible to register signals from such nuclear recoils. As these experiments did not find WIMPs, more sensitive detectors were needed. One possibility was using xenon as a detector material. In the early 2000s, different groups of researchers set up xenon detectors, in deserted mines of the UK and the US. Aprile and her international collaborators (around 150 of them) sought out a laboratory carved out of the rocks 1400 meters below the surface of the Gran Sasso Mountains in central Italy (*Laboratori Nazionali del Gran Sasso*). The core of their detector is a stainless steel vat filled with xenon. The XENON10 held 10 kilograms of xenon. It was followed up by the XENON100 and the XENON1T—holding 100 and 1000 kilograms respectively. The current, fourth, iteration is called the XENONnT, where ‘nT’ stands for *n tonne*, with eight metric tons of xenon (Figure 1). And even though no WIMP has been detected over all these years, the hope that it might happen has remained alive. In an interview she gave in 2016, Aprile declared: ‘It’s not a job. It’s a quest.’ (Sokol, 2016)

## A clean experiment works

As we asked our informants what cleanliness means for the XENON detector, the most succinct answer we received was this: ‘A dirty experiment doesn’t work, a clean experiment does.’ In this case, then, cleanliness allows for functionality. To provide a sense of what it is for a detector to work, we share an outline of the technical details we learned from our informants.

**Figure 2.**
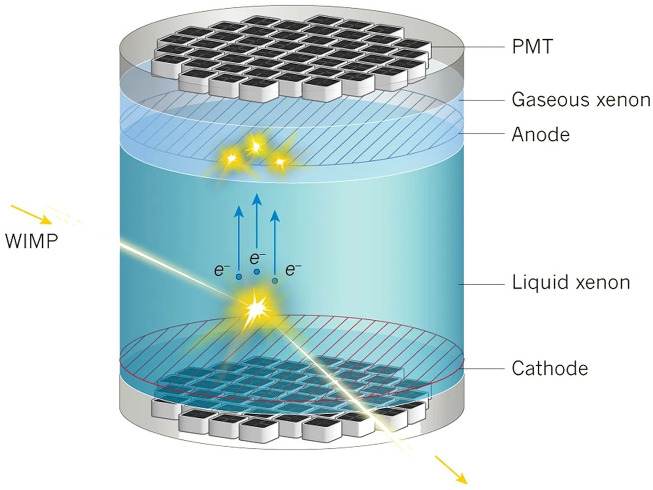
A representation of a hypothetical WIMP detection by the XENON experiment. The interaction of a WIMP will create two signals, one from the initial collision and another from the electrons that drift upward in the liquid xenon. The cathode and anode provide a strong electric field that makes the electrons drift upward. PMT is a ‘photomultiplier tube’ that is able to detect the emitted light. *Source:* Adapted from Ji (2017).

At the core of the XENON detector is a vat that holds liquid xenon, cooled to minus 100 degrees Celsius. Due to the large size of xenon atoms, the expectation is that, if there are many in a vat, sooner or later a WIMP should bump into one of them. A collision would then make the xenon atom scintillate—a property of xenon—causing the emission of a photon. Such photons would be detected by *Photo-Multiplier Tubes* (PMTs) surrounding the vat. The collision would also produce free electrons, and strong electric field would drift these electrons upwards so that at the top of the vat, they would hit gaseous xenon atoms, which would again release photons, that the PMT sensors are also able to detect. The combination of the two signals should make it possible to locate and characterize the interactions between the xenon atoms and WIMPs being sought (see Figure 2).^6^ The expectation was that the event of a WIMP interacting with xenon atoms would occur a few times every year.

Crucial to ensuring the experiment’s sensitivity to WIMPs is avoiding what the researchers call *background*, non-WIMP particles that strike xenon atoms and thereby unduly light up the detector. Backgrounds, as Knorr Cetina (1999, pp. 50–51) notes in her case, are previously understood physics entities that keep popping up but are considered *uninteresting*. If background signals are sufficiently different in energy from what is expected of WIMPs, they can be dismissed as irrelevant.^7^ However, if a background signal too closely mimics what is expected of WIMPs, it generates false positives and thus spoils the experiment. This means that the detector has to be kept materially clear from any WIMP-mimicking background. A first possible variant of such backgrounds, are outside particles like energetic cosmic rays. To shield the experiment from these, the xenon vat is located under 1400 meters of solid rock and hung in a giant water tank. This location, however, does not protect the vat from background emerging from radioactive materials within the lab. All kinds of materials may be radioactive. Our informants joked that the bananas they snack on might well ruin ‘low-background experiments’ like XENON: Bananas contain potassium, rich with radioactive isotopes that could mimic WIMPs. The extreme sensitivity of the detector to radioactive materials prompts intricate efforts to ensure the cleanliness that we describe below.

These efforts are also meant to prevent the free electrons—released if a WIMP were to interact with a xenon atom—from disappearing or being ‘eaten’, as the experimentalists put it. In the words of Aprile: ‘We use phototubes to see photons, but we still rely on the electrons which are liberated by the energy—released by let’s say a WIMP [when it collides with a xenon atom]—and those electrons get easily eaten.’ Another researcher explaining cleanliness to us says that the xenon should be ‘free from molecules like oxygen, which love to eat charges, electrons. It should be free from electronegative impurities.’ The problem with electronegative impurities, then, is not that they *blur* signals (like noise—a more prominent problem in a lot of other experiments), nor that they *mimic* signals (like radioactivity in the XENON experiment).^8^ Instead, they *erase* signals: electronegativities come with the risk of signal destruction. It is no mean feat to prevent this. Molecules that suck up electrons are ubiquitous, they include oxygen, water, and carbon dioxide. No wonder, then, that when we interviewed Aprile, she sighed, ‘That’s been all my life, purifying xenon so that you can get these electrons to live long.’

## Making XENONnT the cleanest place on earth

**Figure 3.**
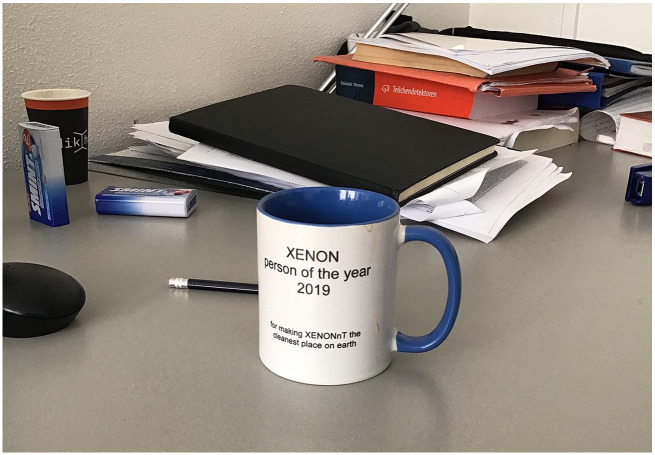
Tobias’s coffee mug. *Source:* Photo by the authors.

What it takes to ensure the cleanliness of the XENON detector is beyond the purview of most dark matter researchers, who engage with theoretical problems in offices, using data that come from elsewhere. When we asked these people about cleanliness, they raised their eyebrows and look nonplussed.^9^ As one of our informants jokingly put it, ‘For them, the XENON experiment is simply a block of xenon.’ Perhaps that is why Tobias seemed rather glad to talk with us. Finally, someone was interested, not just in the end result of his work, but in all the effort it took to get there. On his desk, he had a stained coffee mug, a gift from his colleagues, with the inscription: ‘Person of the year 2019, for making XENONnT the cleanest place on earth’ (Figure 3). But this sign of recognition did not quite counterbalance what felt like an underappreciated, taxing job: that of leading the ‘cleaning campaign’.^10^

The first step in ensuring a clean detector was the procurement of clean materials. To begin with, the xenon inside the vat had to be uncontaminated—a locally salient word for ‘clean’—by radioactive isotopes. The purest possible xenon was bought from a specialist facility that industrially distills xenon from the air. Their procedures were insufficient to eliminate trace amounts of other gasses present in the atmosphere. Particularly hard to eliminate is the isotope krypton-85, ironically first released into the atmosphere through nuclear weapons testing. To deal with this, the experimenters further cleaned their xenon with dedicated filters. A further concern was that the stainless steel, copper, and Teflon that made up the detector had to be radiopure (uncontaminated by radioactive isotopes) as well. Tobias explained, ‘Each material we use, we take samples and we measure how much radiation comes out.’ These measurements fed into decisions, not just about which materials to use, but also about which companies to work with. The main problem here was that the version of *clean* relevant to the experiment diverged a great deal from the version of clean relevant to other practices.

Take the stainless steel, used for the vat and for mounting the entire detector construction. Radioactivity may enter steel throughout its entire production process. Tobias explained, ‘Say, if you put [the steel] in a bath or leach it with something which has high radioactivity, then you have it.’ In addition, steel manufacturers often make use of recycled steel. ‘Imagine recycling an old nuclear submarine’, collaborator Ben remarked. But if radioactivity may contaminate steel accidentally, it may also be added on purpose. To quote Tobias again, ‘There are also companies that mark their stainless steel with radioactivity. This is common because it’s easy. … It’s easy, you can mix it in, it’s not dangerous because it is lower radioactivity and it’s easy to measure. But, of course, this is death for us.’ The amount of radioactivity that kills (hinders the working of) the detector is tiny, much lower than the amount considered to threaten human health.^11^ Ben added, ‘We’re really talking about homeopathic amounts of radiation.’ To give you an idea, the steel in XENONnT radiated approximately 10 micro becquerels (µBq) per kilogram, while a banana typically radiates about 15 becquerels (Bq) (Ball, 2004). This makes for a difficult conversation with the steel manufacturers. Ben had to deal with them directly: ‘Those fuckers who make the steel—who sell us the steel pipes—we asked them if it wasn’t radioactive. “Yeah, of course it is not radioactive.” Then we want to cut off a piece and measure it.’ It is a wonder that they found a suitable company at all. Tobias noted, ‘Imagine, you need to find a company willing to give you a sample and wait until you finish your measurements without selling the particular batch of stainless steel you are investigating. … You know, we are not good customers! We want to have the best and we want to have them waiting for us—and then we take only tiny amounts.’

Because the requirements of the experiment are so idiosyncratic, some of the equipment is built jointly with the manufacturers. This is the case for the sensors meant to detect the photons—photomultiplier tubes (PMTs). These are co-produced with a specialized Japanese company (Aprile et al., 2015). The XENON physicists first test all the materials that go into the PMTs, and then the company optimizes them and builds the sensor. But as collaborator Sandra put it, ‘We screened it again—the manufactured product. And then you discover things you didn’t discover in the materials.’ It turned out that some of the sensors held radioactive silver isotopes that originated from the 2011 Fukushima nuclear disaster. Sandra added, ‘They used some materials that we didn’t screen, so in the end, even if you think you screened all of the materials individually, you cannot trust the product. They may have used some seal that they didn’t tell you about, or something.’

Once the materials are procured and components such as the PMTs have been assembled, the onsite cleaning starts. Tobias explained, ‘So, now you have selected all your materials. They have been produced wherever. And now you ship them to LNGS [Laboratori Nazionali del Gran Sasso] because you want to build your detector. So, now you need, of course, to make sure that you don’t add too much dirt into your detector.’ Before assembling the current XENONnT detector, all the separate parts were cleaned by a provisional cleaning team, which included graduate and undergraduate students flown in for a few weeks to the Gran Sasso laboratory. What did they do? Tobias answered, ‘It’s funny, because you might think this is super fancy. But no, the first thing is that you may be standing there with the water gun and you blow the coarse dirt off. Because a lot of parts come in, like, completely, completely disgusting.’ The term ‘disgusting’ calls up a gut-felt aesthetic dirtiness, but the dirt that is blown off—such as grease, lubricants, and dust—also disrupts the experiment: They contain plenty of *electronegativities* that risk eating the signal, and thus destroying it.

**Figure 4.**
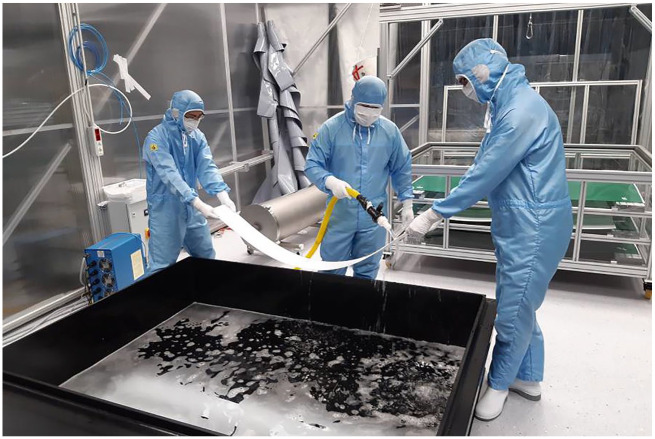
The cleaning of a PTFE sheet in the cleanroom of the XENON experiment at the Gran Sasso laboratory. For this picture to be taken, the camera had to be cleaned in the anteceding ‘grey room’ and kept in a plastic bag. *Source:* Photo from the collection of Tobias.

Once precleaned, the parts were carried into the dedicated cleanroom. This is a five-by-nine-meter space located above ground, away from the main experimental hall situated deep within the mountain. After being wiped with ethanol wipes in the first ‘grey room’, the materials were washed with soap in a sonic bath, rinsed with deionized water, and passed through several acid baths in the cleanroom (Figure 4). For different materials, this was done in different orders. Tobias continued, ‘So, you deal with cubic meters of acid, and you should safely, and in clean conditions, clean this, and clean that. And then pack it in special plastic, store it somewhere, so that it doesn’t recontaminate.’ None of these steps are straightforward. While there are standards for regular laboratory cleanrooms—such as those of geochemistry described by Gieryn (2018)—the XENONnT detector is a one-off device. As Tobias said, ‘Everything, everything is somehow … I mean it’s improvised.’ Some parts were too large to fit through the cleanroom door. A special entrance was made for them; however, this introduced the risk that unfiltered air would enter and contaminate the cleanroom. Some parts turned out to have loose plastic fibers, but improvised attempts to cut these fibers away with a knife risked adding more, rather than less, plastic particles. Some parts were put in the wrong acid baths and destroyed in the attempt to clean them. And, as one student recalled, some humans set the cleanroom’s aerosol alarms off with their flatulence.

## Solving one problem, creating another

That the cleanliness of the detector is difficult to achieve is aggravated by the fact that every act of cleaning risks introducing additional contaminants. For example, the acid in the acid bath may be soaked up by the materials it was meant to clean. These materials may also be contaminated by water or trap air from the cleaning room, including its electronegative oxygen. And then there was the issue of the soap. When Tobias asked industrial cleaning experts about which soap to use, they recommended one that was highly alkaline. This is indeed appropriate when you seek to remove the sticky dirt from surfaces like floors, but it is very high in potassium—not a concern in buildings, but problematic for a dark matter detector. Tobias remarked, ‘So, then you have a bottle of soap, which radiates kilo becquerels of gammas due to the potassium.’ That is not ideal, ‘I at least get a bad feeling saying, oh, I want to have a low background experiment, but then I use a detergent that radiates like hell.’ Hence, like the materials used to build the detector, potential soaps were also carefully tested: ‘We screened all these samples of soap. For some of them we even simulated the cleaning, and measured before and after to see if there were residues on the surface—you know, you don’t want to contaminate during the cleaning. … I mean, it could be that you want to do something good but in the end, you make it worse.’

Once the detector was assembled, the cleaning was still not over. Gasses caught up in the materials of the detector, mainly water vapor and oxygen, could come out again during the experiment (a process called *outgassing*) and act as electronegativities. Teflon is especially problematic in this respect, it is ‘like a sponge’, sucking up soap and air. Yet when copper filters were developed in the hope of purifying the xenon of such molecules, radon gas appeared to seep out of the copper. Radon was a more general and persistent impediment for the experiment. As it is a *noble gas*, radon does not bind to other elements. Hence, it gets easily sucked up by materials like Teflon, but can just as easily be released from them. It exists in trace amounts in the air and has radioactive isotopes, including radon-222, that can decay. This becomes a never-ending source of potential false positive signals. In Tobias’s words, ‘Continuously, this radon comes out of your material. It’s not that you clean your xenon once and then it’s done, because it’s always coming, permanently.’

In the XENONnT experiment, the aim was to reduce the radon emanation background to less than one micro becquerel per kg of xenon—that is, one radon atom for every 10^21^ xenon atoms. This is ten times less than what was achieved in the previous experiment, XENON1T. It amounts to a reduction of the typical amount of radon in atmospheric air by a factor of 10 million—quite an achievement. The experiment uses several filters and machines to purify the xenon, e.g., from krypton. Radon was their hardest challenge. Tobias explained, ‘We were looking for a very good [filter], but it was always too dirty. The filter itself was emanating too much. Each material has this problem, that it will emanate radon. So, at some point, you reach a level where filtering is not going to work anymore.’ As an alternative to filtering, the experimenters built a radon distillation system. Eventually this turned out to be succesful, but in the first run of operation it made things worse. It contaminated the xenon with large amounts of electronegative impurities. As Ben put it, ‘It’s a shame that the machine that should get out the radon screws up the quality of the xenon.’^12^

At this point, the experimenters knew that the new distillation system increased the amount of electronegativities, but they did not know how this had happened, and what exactly the contaminants were. Lack of knowledge systematically plagued them. ‘Nobody tells you what to do. Nobody cares about *this* cleaning, on *this* level’, Tobias said. He would have hoped for data on which he might have based his decisions, but there was no data. Neither on how to clean nor on how to evaluate all the cleaning work. ‘[Finding] publications and proof of [cleaning procedures]—very hard. Very hard.’^13^ When asked how much his cleaning had improved the sensitivity of the experiment, Tobias sighed, ‘Of course, we tried to improve things but in the end, I cannot tell you … I mean, we tested our procedures on samples. But who knows if the samples are exactly the same as our detector materials? … I cannot say how much it helped. I cannot tell you. I really cannot tell you.’

## It will stop eventually

**Figure 5.**
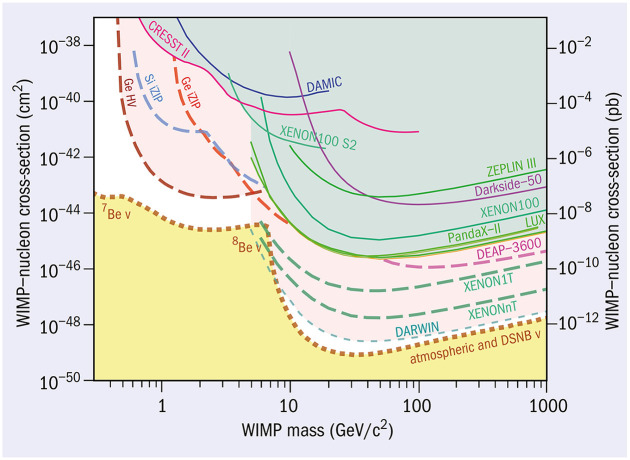
WIMP exclusion plot. The diagram shows a space made up of the potential WIMP cross-section and its mass. (The cross-section says something about the chance of the WIMP to interact.) All the lines represent the results of experiments: the solid one of previous experiments, the dashed one of the experiments to come. The lines show the upper limits of the WIMP size: Everything above the line has been excluded by that experiment. It is the space where the WIMP does not exist. With every experiment, the white space in which the WIMP can still ‘live’ becomes smaller. The yellow region is the neutrino floor: in this region, signals from neutrinos start to dominate, rendering the WIMP practically invisible. *Source:* From Baudis (2017).

‘The history of dark matter detection is a history of cleaning’, a professor involved in a different experiment told us. As physicists built bigger dark matter experiments, the challenge has always been to create cleaner and cleaner environments. This means that, since the late 1980s, the sensitivity of WIMP detectors has increased exponentially: Every new generation was able to pick up signals of ever smaller particles, with ever lower cross-sections (see e.g., Cushman et al., 2013). However, as we already mentioned, to date no WIMP has been detected. Each successive experiment, including the XENONnT experiment, has only been able to assert what—with a confidence level of 90%—a WIMP is not. Hence, knowledge has been generated, but it is what Knorr Cetina (1999) calls *negative knowledge*.^14^ Again and again, this negative knowledge limited the WIMP parameter space—the space in which the WIMP, qua mass and cross-section, might ‘live’. The experimentalists represent this in ‘exclusion plots’ (see Figure 5 for an example).

As the experimenters pushed their experimental setup, the cross-section of the hypothetical WIMP has continued to decrease. But just minimizing the target of one’s quest is not particularly rewarding. Aprile noted: ‘When you look back at the history of the project … at the time the XENON10 started, our theory friends would tell us, “Oh, you’re going to see this. It’s right there.” Then as we explored more and excluded the region they had predicted, they would refine the model and tell us we would see WIMPs at the next turn. And then nothing still, but they always found a way to explain why the WIMP was not there and how you needed to improve sensitivity. This is what we continued to do. Another order of magnitude and another order of magnitude. We’re talking almost five orders of magnitude since XENON10! It’s a tremendous achievement, but sadly we are still missing a signal.’ (Aprile, 2021b)

Might the enduring absence of a signal be due to the residual uncleanliness of the detector? Who knows. The experimenters know that the leftover krypton in the xenon is only parts per *quadrillion* and that after successfully installing the distillation system the radon level has been reduced to 0.9 micro becquerel per kilogram of xenon (Aprile et al., 2017; Aprile et al, 2025). They published an entire paper about the ‘material radiopurity’ of the experiment (Aprile, Abe, Agostini, Ahmed Maouloud, Alfonsi, et al., 2022). But even with the detector conforming to all pre-established norms of cleanliness, nobody can tell whether the detector is clean enough. If a WIMP were found, this would count as convincing proof that the detector was adequately clean. As long as no WIMP is found, it remains impossible to tell what ‘adequately clean’ entails for a XENON detector. Years ago, Collins (1985) introduced the notion of *experimenter’s regress* to contend that in the case of a novel, as-yet-undetected phenomenon, a circular logic emerges: A given signal either proves the existence of that phenomenon or is a false positive—and yet which of those options a signal is depends on whether the phenomenon exists. By analogy, we might say that what we encounter here is a case of *cleanliness regress*: You only get a result if your detector is clean enough, but you only know that your detector is clean enough if you get a result. With the ontological question of whether WIMPs do or do not exist remaining open, normative reassurance about the detector’s cleanliness, too, stays out of reach.

Tenaciously, the collaboration goes on. The researchers are now planning yet another, larger experiment, called DARWIN (DARk matter WImp search with liquid xenoN). This is meant to use 40 metric tons of xenon and is projected to be sensitive to particles with a cross-section of 10^−49^ cm^2^, yet another order-of-magnitude increase in sensitivity beyond the current XENONnT limit. The collaboration refers to it as ‘the ultimate dark matter detector’ (Aalbers et al., 2016). It is ‘ultimate’ because at some point increasing sensitivity any further makes the detector sensitive to neutrinos. These particles are released in common nuclear reactions throughout the universe: The sun produces neutrinos, and cosmic particles entering the atmosphere of the earth produce neutrinos. Our informants assert that with a xenon experiment, it is all but impossible to distinguish between neutrinos and the hypothesized WIMPs. Hence, the experiment is bound to hit upon a limit that they call the *neutrino floor*: ‘the point at which dark matter signals become hidden underneath a remarkably similar-looking background from neutrinos’ (O’Hare, 2021, p. 1).^15^ Or, as one of the collaborators put it, ‘This is an experiment where you don’t see anything until you hit a background … A lot of backgrounds we can remedy—for example by cleaning and by properly handling our xenon. But yes, eventually there is a source of neutrinos where we cannot get around. And yes, then you’re done and over with.’ Aprile regrets the fact that her quest cannot go on forever: ‘It will stop eventually, with neutrinos from the sun, or a signal from neutrinos at least. Then, you will never be able to tell if it is a signal from neutrino, or if it’s a signal from dark matter.’^16^

Ideally, experiments end when there is a result, when ‘evidence is elevated from a hint to a demonstration’ (Galison, 1987, p. 1). The xenon-based experiments, however, may end in limbo with the detector hitting the neutrino floor. Detecting neutrinos is its own kind of achievement, but it puts an end to the dream of detecting WIMPs.^17^ Thus, after all the intricate cleaning, reaching the neutrino floor will shift the xenon dark matter detector from *exceedingly clean* to *irredeemably dirty*. The normative goal of cleanliness will forever remain out of reach, making the ontological question of whether WIMPs exist impossible to answer. Aprile dreads the moment: ‘If you’re an experimentalist you want to measure something. And when you find nothing, there’s no events other than background—yeah, it’s, well, definitely it’s not a good feeling.’

## Other dark matter candidates, other versions of clean

As the search for WIMPs is hitting upon the limits of what Radder (2012) would call its ‘material realization’, it is also hitting upon the limits of its social support among the larger community of dark matter researchers. In 2010 already, a prominent dark matter physicist published an article in *Nature* (Bertone, 2010) with the title ‘The moment of truth for WIMP dark matter.’ He writes (p. 389), ‘Either we will discover them in the next five to ten years, or we will witness their inevitable decline.’ When the ten years were almost over, in 2018, the same author signaled ‘a growing sense of “crisis” in the dark-matter particle community’ (Bertone & Tait, 2018, p. 51). Evidence for the existence of supersymmetry—one of the theoretical backbones that suggested WIMPs as the most likely dark matter candidate—also remained lacking. ‘[It] raises the possibility that the natural WIMPs may have been nothing more than an attractive red herring’ (Bertone & Tait, 2018, p. 51). In line with this, some students who had helped with the cleaning were doubtful about the salience of their work. One of them asserted that, ‘The quest was narrow-minded as it kept searching for only one thing, very hard.’ Another, wondered hesitantly, ‘What if we are going about searching for dark matter in a totally wrong way?’

Prompted by such sentiments, various groups of dark matter researchers have recently shifted their priorities, and started new experiments, using new techniques, to search for dark matter candidates other than WIMPs.^18^ These experiments all require cleanliness, but as they engage with different ontological repertoires, the relevant version of cleanliness varies each time. Take the experiments searching for *axions*—hypothetical ‘ultra-light’ dark matter particles named after a laundry detergent because their existence would ‘clean up’ a sticky theoretical problem.^19^ The technology that is used to detect axions includes ‘resonating cavities’ that are sensitive to the effects of electromagnetic fields and vibrations. Hence, keeping these instruments clean involves technologies that shield them from electromagnetic and vibrational disturbances emerging from cooling machines, pumps, amplifiers, and even local radio communication.^20^ Other physicists take it that a vast number of *primordial black holes*, formed in the early universe, might account for the missing dark-matter-masses. To find evidence for black holes, they use highly sensitive lasers and mirrors that allow them to register their gravitational signatures. The lasers must avoid producing light at unwanted frequencies, while the mirrors get disturbed by vibrations caused by things such as heat and seismic noise from vehicle traffic.^21^

In both *axion* and *black hole*-directed dark matter research, cleanliness is essential, but neither of the experiments just mentioned is bothered by the radioactive isotopes or the electronegative molecules that disturb the working of the XENON detector. This is clearly revealed by the location of one of the LIGO gravitational wave observatories, which is looking for signs of black holes. It is built in Hanford, Washington, U.S., on the site of a dismantled reactor that once produced plutonium—including the plutonium used for the atomic bomb that was dropped on Nagasaki. This site swarms with radioactive isotopes (e.g. Brown, 2013; Cram, 2023). While their ongoing decay has turned out to be disturbing for human health, it would be anathema for a WIMP detector. For a gravitational wave detector, however, Hanford is a great location. The radioactivity does not interfere with the working of the detector’s lasers and mirrors; instead, it helps keep noisy humans and their even noisier vehicles at bay—which are dirty disturbances for the LIGO detector (Nichols, 2024). The ontological repertoires in which both experiments fit their hypothesis about what ‘dark matter’ *is* are different, the equipment they use differs accordingly, and along with this comes a huge gap between the norms by which they evaluate cleanliness.

## They aren’t in an ivory tower, they are in a society

**Figure 6.**
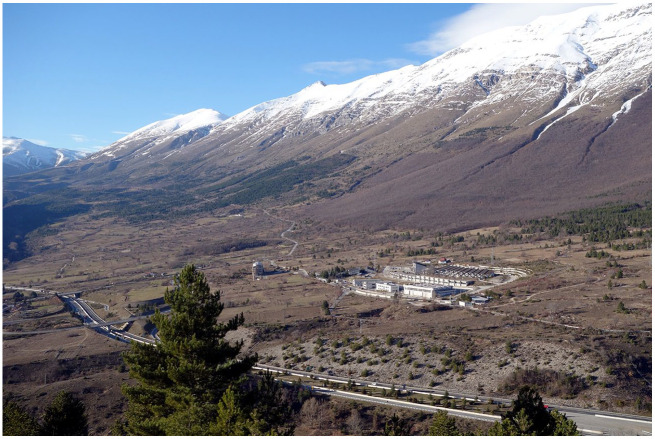
The Gran Sasso National Laboratory above-ground facility in the Gran Sasso National Park. *Source:* Author: TQB1 License: CC-BY-SA 3.0.

The fact that WIMP detectors and gravitational wave detectors engage with different ontological repertoires and depend on different versions of cleanliness does not give rise to disputes, let alone clashes. However, in situations where practices materially interfere with each other, differences may turn into tensions. To illustrate this, let us return to the cleaning of the XENONnT detector. The Gran Sasso National Laboratory is located within the Gran Sasso National Park, a designated nature reserve (Figure 6). This protects the site from human intruders and other potential disturbances. However, it introduces a further kind of cleanliness that Tobias and his team had to take into account, next to that of the detector: *environmental cleanliness* as per the regulations of the local government.

In 2002 an earlier experiment taking place in the Gran Sasso National Laboratory accidentally spilled detector fluids that were toxic—disturbing and thus dirty—for living organisms. This turned into a scandal as the toxins threatened to seep into the aquifer underneath, which provides drinking water for the villages over a wide neighboring area. In response, the local government came up with a set of requirements: Floors had to be resealed and safety officers had to be appointed. When there was a further spill of harmful liquids in 2016, local environmental groups protested fiercely. Several chief officers of the lab were prosecuted for ‘negligence and imprudence’ (Cartlidge, 2018). As a result, two major experiments working with large amounts of toxic liquids had to shut down. The president of the environmentalist *Abruzzo Ornithological Station* spoke in support of this prosecution. He said to a journalist, ‘I understand that the scientists want to do their experiments but they are not in an ivory tower, they are in a society’ (De Sanctis, in Cartlidge, 2019, p. 12).

**Figure 7.**
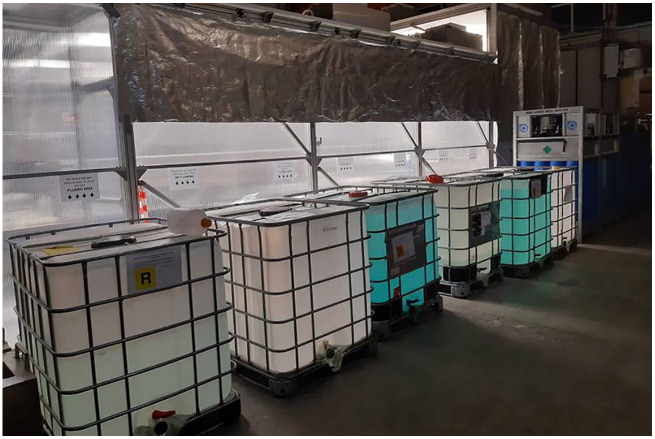
Chemical waste containers meant to collect the used soap and acid solutions after the cleaning procedures. The containers are provided and managed by the LNGS environmental service. *Source:* Photo from the collection of Tobias.

For the scientists using the Gran Sasso National Laboratory, being in society now meant that their experiments risked ending with neither a demonstration, nor in limbo, but with prohibitions and prosecutions. To prevent this, the Gran Sasso laboratory installed a *Servizio Ambiente*, an Environmental Service, that arranges, as their spokesperson put it, ‘all procedures, regulations, problems, and responsibilities of all the phases of the experiment’. The service communicates with local authorities, monitors the wastewater, and arranges trucks that carry out various kinds of waste (Figure 7). Because, as the spokesperson stated, ‘We have to deal with this particular reality in our lives.’ This so-called particular reality came with its own normative requirements for the ‘cleaning campaign’.^22^ All the cleaning materials used to ensure the particular cleanliness relevant to the XENONnT detector had to be packed up and carted out. This included acid baths, ethanol wipes, thousands of synthetic tissues, and even all the water that was used. Added to that were piles of face masks, hairnets, and clean suits, as everyone involved put on a fresh set when reentering the cleanroom after a break, about five times a day. Tobias prepared for all these materials to be taken out by the trucks that the *Servicio Ambiente* would arrange. When we asked him where it went, he said, ‘My heart must bleed there. I don’t know. For me, this was picked up and disappeared.’

To ensure the continuity of the experiment, potential tensions between the cleanliness relevant to the detector and the very different cleanliness relevant to the National Park are kept in check. However, something stays out of this picture: the environmental cleanliness of the places where the discards of the laboratory end up. Tobias does not even know where they are—‘my heart must bleed there’. And it is not just that avoiding the disturbance of living organisms here comes at the cost of living organisms there. It is worse: The environmental regulations meant to protect the reserve aggravate problems in other sites. For instance, in order to avoid another spill into the aquifer, it is forbidden to use acids, aggressive soaps, and the like, in the underground lab. This is why the cleanroom is above ground, where things are easier to manage. However, this means that there is a distance to cross between the cleanroom and the lab. To avoid contamination along the way, every part of the detector-to-be is wrapped in a special sealing plastic. Hence, the attempt to protect the National Park from acids and soaps means that some other environment is saddled with a heap of extra plastic. Another twist is that environmental regulations in Gran Sasso prohibit the onsite use of equipment designed to measure the radiopurity of the xenon, as it contains large amounts of toxic mercury. As a way out, xenon samples are packed and travel 900 kilometers by air, from central Italy to be tested in Heidelberg, with the same type of equipment. In this way, the risk of spilling mercury is not avoided, but displaced to another site—while the extra travel generates additional CO_2_ and soot—disrupting the function of living organisms elsewhere. All of this means that ‘the cleanest place on earth’ can be quite dirty if you are not concerned about the reality of WIMPs, but rather about the ‘particular reality’ of a national park or some other natural environment. As long as scientists translate ‘being in society’ into deferring to governmental rules, they delegate out, rather than address, the question which of these realities to care for (Puig de la Bellacasa, 2011).

## To conclude: Dirt is a disruptor

Tobias received a mug for having made the xenon detector ‘the cleanest place on earth’. But the detector was only *the cleanest* on its own terms: It was exceedingly low on radioactivity and electronegativity. For a xenon detector, radioactive contaminations and electronegative molecules figure as dirt; they are disruptors and make detecting the hypothetical WIMPs impossible. Hence, with a lot of effort they had been cleaned away. In most other settings, however, these specimens of dirt are not particularly interesting, they disrupt nothing; they are not ‘dirt’ at all. This already applies to other dark matter experiments: neither axion searches nor black hole inquiries are bothered by small (let alone minute) amounts of radioactivity or electronegative molecules. These experiments depend on other kinds of cleanliness. For environments both near and far, the exceedingly low levels of radioactivity and electronegativity targeted by the XENONnT experiment’s ‘cleaning campaign’ would be irrelevant, however, the very practices employed to achieve them, risk to (in the case of the Gran Sasso National Park) or are bound to (in the case of undisclosed environments elsewhere) be polluting. This confirms that *clean* is not simply a quantifiable *good* that *cleaning* helps to achieve: It is a valuation that only gains its salience in relation to a specific ontological repertoire—while activities meant to achieve one kind of cleanliness, may unduly spoil another.^23^ Hence, what is at stake in the laboratory is not just knowledge and its social and material preconditions, but also realities—in the plural—and their situated qualities.

That ontology and normativity are intertwined means they can remain jointly elusive—in our case they do. Here, then, we learn that theoretically dreaming up an ontological repertoire that enacts WIMPs as *potentially real* does not suffice to materially and practically establish them as *actually real*. For this to be the case, a signal that fits with WIMPs hypothetical traits has to be caught. So far, this has not happened. And with the ontological question about whether WIMPs exist remaining open, so, too, remains open the normative question as to whether the detector was clean enough. The further twist, tragic and ironic, is that more cleaning makes xenon detectors sensitive to neutrinos. As the signals of these neutrinos are bound to mask the signals of potential WIMPs, they disturb the detector’s ability to function. This means that with more cleaning, the detector turns from extremely clean to irredeemably dirty. And with the normative goal of adequate cleanliness out of reach, it also becomes impossible to answer the ontological question of whether WIMPs exist. In this case, then, both WIMPs and cleanliness—the locally pursued ontology and the locally salient normativity—are jointly and interdependently elusive.

Meanwhile, the practice of cleaning the detector involves a lot of ‘cleaning materials’ that, once used, are discarded. As Tobias dutifully abides by the instructions provided to him by the laboratory’s *Servicio Ambiente*, the discards involved do not pollute the Gran Sasso National Park where the XENON experiment is located. All discards—including the water—are trucked out and go elsewhere. We did not trace where they ended up and what they may or may not have polluted there. For now, we just conclude that different versions of cleanliness are not only distinct, but may also be in tension with one another. In this case, they are—since caring for the cleanliness internal to the detector leads to the externalization of environmental mess. To prevent this mess from polluting the local nature reserve, they are shipped out to other environments, elsewhere, adding a few extra discards in the process.

Scholars straddling STS and discard studies have already flagged that the environmental dirt created by laboratory research deserves far more attention (Liboiron, 2021; Liboiron & Lepawsky, 2022). This we wholeheartedly underscore. While our case is highly specific in some respects, it illustrates once again a far more general conundrum: The very practices meant to care for a particular version of cleanliness here generate discards that elsewhere figure as dirt. This is true far beyond research laboratories. Scrubbing the dirty floors of buildings with alkaline soaps also means that water downstream gets loaded by stuff that dirtied those floors plus stuff contained in the soaps. Likewise, caring for hygiene in hospitals or households creates impressive amounts of environmental mess. Etcetera. Hence, the questions we end with: Yes, cleanliness may be a good. But which cleanliness is good for what? And if caring for something that is appreciated as clean here, creates what acts as dirt there, how to handle the tensions that ensue? When, or to what extent, is it acceptable for a quest meant to find hypothetical entities to spoil the lives of organisms, existing alright, but fragile? In other words: Which goods deserve to be cherished, and which realities should we care for?

## Notes

1. In her study of high-energy physics, Traweek (1988, p. 39) attends to contamination as something that risks to travel from inside to *outside* the laboratory. See for a recent case of fear of radioactive contamination, and for ways in which this was smothered after the Fukushima accident, Takahashi (2024).

2. Also inspired by Douglas, but less about cleaning and contamination, Sims (2005) shows how, in a Los Alamos laboratory community, concerns with safety harbor ideals of social and material order.

3. Mol (2020) argues that Douglas is concerned with *tidiness*, rather than with *stickiness* or things that are unclean in that they smell or taste *dirty*. If dirt is a disruptor, then what clean is does not depend on what is order in a context, but what is working in a practice. For a case that clearly shows that what is disrupted by dirt does not depend on (a given) order, but on (particular) stakes, see Pols’s (2006) analysis of *washing* in long stay psychiatric clinics, in which different concerns with cleanliness went hand in hand with different versions of ‘citizenship’.

4. Earlier STS inquiries into ontologies-enacted made contrasts between *versions* of reality, but did not go to the edge of ‘ontological doubt’ as to whether this or that entity *exists*. For example, Mol and Berg (1994) contrast clinical, statistical and pathophysiological variants of ‘anemia’, that come with different ways of assessing whether *this person* has ‘anemia’, but that all three presume the existence of ‘anemia’ in *some people*. Akrich and Pasveer (2000) show that ‘the fetus’ is not the same thing/being from one obstetric practice to another, but their study does not include sites where the very existence of ‘fetus’ is in doubt.

5. Early ideas to explain dark matter included hot gas and faint stars. These did not to work out. WIMPs, thought to have formed in the hot soup of the Big Bang, and still crisscrossing the universe, would add the gravitational force cosmologists were looking for, but were also substantiated by the theory of supersymmetry, which fit with the agenda of particle physics. Added to that, the existence of a WIMP-like particle would help to explain why the mass distribution in the universe is not homogenous but lumpy. See e.g. Peebles (2020).

6. This makes XENON a *dual-phase* detector. The *location* of the interaction can be determined from the hit pattern on the PMTs and the time difference between signal 1 and signal 2. The *type* of interaction can be determined from the ratio of the strength of the two signals, s1 and s2. The location of the interaction is important, because at the edges of the vat more background events are expected.

7. In a XENON vat two types of collisions can produce a detectable signal: collisions with the xenon *nucleus* and with the xenon *electrons*. Both interactions create photons with different energies. Although mostly quite distinct, these energy bands can ‘leak’ into each other. A lot of statistical work can discriminate between the bands—but not always. See e.g. Pinel et al. (2020).

8. Fellow scholars kept asking us whether these concerns about contaminants are problems of *noise*. They are not. Noise, for our informants, also exists: It is the static inherent to the workings of the electronic apparatus meant to pick up the photons and convert them to digital signals (electronic, or thermal noise). This echoes Helmreich’s (2016) observations, when he reiterated that gravitational wave detection depends on distinguishing signals from noise: ‘the “noise” is noisy—staticky, buzzy, hummy, a fuzz against which to pick out a clean signal’ (p. 482). See also Knorr Cetina (1999, p. 51). The *backgrounds* of our informants are different: they come from physical processes, like radioactive decay, that cause *events* that can perfectly mimic signals coming from dark matter. These backgrounds, unlike noise, can be controlled, physically cleaned away—at least, in principle.

9. In the ‘journal clubs’ that we (that is to say, the first author) attended, a lot of experimental and theoretical knowledge was exchanged, but the focus was always on results or potential results. Compare also Galison (1988).

10. The disregard for cleaning work neatly fits with what Shapin (1989, p. 561) has called the phenomenon of the ‘invisible technicians’.

11. According to the US National Council on Radiation Protection and Measurement the average annual dose humans receive from cosmic radiation is 0.34 millisievert. In different regions of the world there are different regulations, but, as an example, the US Code of Federal Regulations has set as an occupational limit 50 millisievert a year. The reference to *death* can be understood in relation to Knorr Cetina’s observation that physicist use metaphors that turn dirt into an enemy: ‘The background, thus, can be lived with if it must, but only at a price. The overall metaphor is one of fighting an antagonist with all means possible’ (Knorr Cetina, 1999, p. 126).

12. The failure of the purification system was briefly mentioned in the first publication of nT results: ‘While commissioning this new [radon-removal] system we found that, at the time, operating it … resulted in a drastic drop of xenon purity’ (Aprile, Abe, Agostini, Ahmed Maouloud, Althueser et al., 2022).

13. Again, this is a marked difference with other cases, for instance that of the cleanroom in the field of Metal Isotope Geochemistry studied by Gieryn. In geochemistry there were many such cleanrooms and while they, too, found cleanliness to be elusive, they still had *criteria* for it as well as cleaning *protocols* that made it easy for junior researchers to shift from one lab to the next. See Gieryn (2018, ch. 8).

14. Knorr Cetina (1999) describes negative knowledge as ‘knowledge of the limits of knowing, of the mistakes we make in trying to know, of the things that interfere with our knowing, of what we are not interested in and do not really want to know’ (p. 64). She observes that negative knowledge has a central role in high-energy physics because knowing a phenomenon depends on knowledge of the errors and uncertainties of involved apparatuses and the corrections for these peculiarities. In our case too, limits on the dark matter particle parameter space are liminal knowledge, used as a ‘vehicle for positive knowledge’ (p. 71): One experiment’s limits are the next experiment’s starting point.

15. As of late, physicists started using ‘neutrino fog’ as an alternative term to describe the issue of the looming neutrino background. This terminology is meant to emphasize that neutrinos come to dominate the background gradually and also to leave some hope that it may still be possible to decern WIMP signals. See O’Hare (2021). As we were re-rewriting this text, publications asserting that xenon detectors were being confronted with the ‘neutrino fog’ started to appear, see Aprile et al. (2024).

16. Some physicists are presently designing techniques meant to distinguish between neutrino and dark matter signals, such as directional detectors that determine where the interacting particle came from. We leave out this complexity here, encouraged by the fact that most of our interlocuters, for various reasons, did not believe a directional WIMP detector will ever be realized.

17. In countering contaminations in WIMP detection, sometimes such contaminations are elevated into new objects of study—like in the case of a rare detected tritium decay (Aprile, Abe, Agostini, Ahmed Maouloud, Althueser et al., 2022). Mody, in his study of material scientists, recognizes that in his case dirt is not only destructive, but that there are many ways in which ‘dirt is used positively to create knowledge’ (Mody, 2001, p. 32).

18. There are also researchers who doubt not just WIMPs but the very existence of dark matter, which implies (depends on) revisiting current theories of gravitation. Although this alternative explanation is interesting for many different reasons—historical, philosophical—it goes beyond the purview of the current paper. See e.g., McGaugh (2014) and Martens et al. (2022).

19. Theoretical problems, then, may also be talked about as dirt to be cleaned away—another line to pursue! See Wilczek (2016).

20. ABRACADABRA is one of the experiments looking for axion dark matter signals (Ouellet et al., 2019).

21. For black holes as dark matter, see Green and Kavanagh (2021). And for the sensitivity of one of the relevant detectors, LIGO, Martynov et al. (2016).

22. The materials within the laboratory are not just potentially dirty for organisms living in the environment, but also for the humans working within the lab (Sims, 2005; also Calkins, 2021).

23. In this article we assert that ontological repertoires and norms/valuations are intertwined, but do not delve into modes of ‘intertwinement’. There is more to explore there, such as the notion of ‘evaluation culture’ that MacKenzie and Spears (2014) propose in the context of finance.
